# Genetic characterization of the artisanal mud crab fishery in Myanmar

**DOI:** 10.1371/journal.pone.0204905

**Published:** 2018-09-28

**Authors:** Iris Segura-García, Thu Yain Tun, Stephen J. Box

**Affiliations:** 1 Smithsonian Marine Station at Fort Pierce, National Museum of Natural History, Fort Pierce, Florida, United States of America; 2 Rare, Global Fisheries, Arlington, Virginia, United States of America; National Cheng Kung University, TAIWAN

## Abstract

Fish are important for food supply, especially in developing countries. In Southeast Asia, including Myanmar, the mud crab fishery is an important livelihood that represents a valuable source of income and food to coastal communities. However, the increasing demand for mud crab in domestic and international markets and poor management has generated concern about the status of this fishery across Southeast Asia. In this region, at least four species of mud crab in the genus *Scylla* are recognised but their correct identification and occurrence remain to be fully explained. Relying on accurate taxonomic identification of mud crab species represents the cornerstone of the successful implementation of management plans as life history biology and relative exploitation rates may vary by species due to gear susceptibility. Toward this aim, tissue samples from mud crabs were collected from four fishing communities of the Mergui archipelago, in the Tanintharyi region of southern Myanmar. All crab samples were DNA barcoded for species identification through sequencing. This study is the first genetic characterization of the mud crab fishery in Myanmar and revealed that *Scylla olivacea* was the only species found in the sampled fisheries of the Tanintharyi region. The populations studied across the Mergui archipelago did not show evidence of genetic structure, but gene flow appeared to be limited among conspecifics from neighbouring countries.

## Introduction

Mud crabs in the genus *Scylla* (de Hann 1833) are large edible crustaceans associated with mangroves throughout the Indo-West Pacific region [1,2]. They are a valuable marine food product in domestic and export markets that constitute an important source of income for coastal fishing communities [3,4]. Unfortunately, the lack of adequate management to regulate catches by small-scale fisheries has generate a concern about the status of this fishery. The knowledge of the species molecular identity, composition and population dynamics of the fishing stocks are still scarce, thus precluding the appropriate management actions.

Mud crab species in the genus *Scylla* exhibit high polymorphism in coloration and size, with overlapping morphologies and habitats between species, which has resulted in a controversial taxonomy since the early description of *Cancer serratus* from the Red Sea in 1775 by Forskål [5]. Several attempts to clarify these nomenclatural and identification difficulties have been conducted locally in Southeast Asia including the Philippines [6], Vietnam [7], and Japan [8]. These studies led to a revised taxonomy of mud crabs in the genus *Scylla* across the Indo-Pacific, using external morphology and genetic variations, to recognize four currently accepted non-hybridizing species; *S*. *serrata* (Forskål 1775), *S*. *tranquebarica* (Fabricius 1798), *S*. *olivacea* (Herbst 1796), and *S*. *paramamosian* (Estampador 1949) [5,9]. Local evaluations across Southeast Asia were also conducted following Keenan et al. (1998) taxonomic review to reveal the actual species occurring locally in Vietnam [10], China [11], Bangladesh [12], Thailand [13], Japan [14], and India [15].

In Myanmar, “crabbing” is a flourishing practice mostly practiced throughout the mangroves and tidal flats of the Ayeyarwaddy delta in Rakhine state [4] and the Tanintharyi region in south Myanmar. Despite their commercial importance little is known about the occurrence of mud crab species in the fishery, which is a major constraint for stock assessment, the development of aquaculture and regulation of the fishery [16]. The correct identification of species occurring in Myanmar and their contribution to the mud crab fishery represent a critical starting point to characterize and to promote appropriate regulations base on species biology. In this regard, DNA barcoding provides a well-established method for species identification by using standard fragments of the mitochondrial DNA *cytochrome oxidase subunit I gene* (mtDNA *COI*) to detect differences among species [17]. This method has been successfully used to identify a broad range of marine organisms (see [18]).

The aim of the present study was to identify the species of harvested mud crab from the fishery communities in the Mergui archipelago using DNA barcoding, and to investigate the genetic diversity and population structure across the Mergui archipelago, in the Tanintharyi region, the largest crabbing region in Myanmar. We resolve the taxonomy of locally harvested mud crabs and population structure based on mtDNA *COI* data, as critical information contributing to the broader understanding of the mud crab fishery in the region.

## Method

### Sampling and DNA extraction

**Fig 1:**
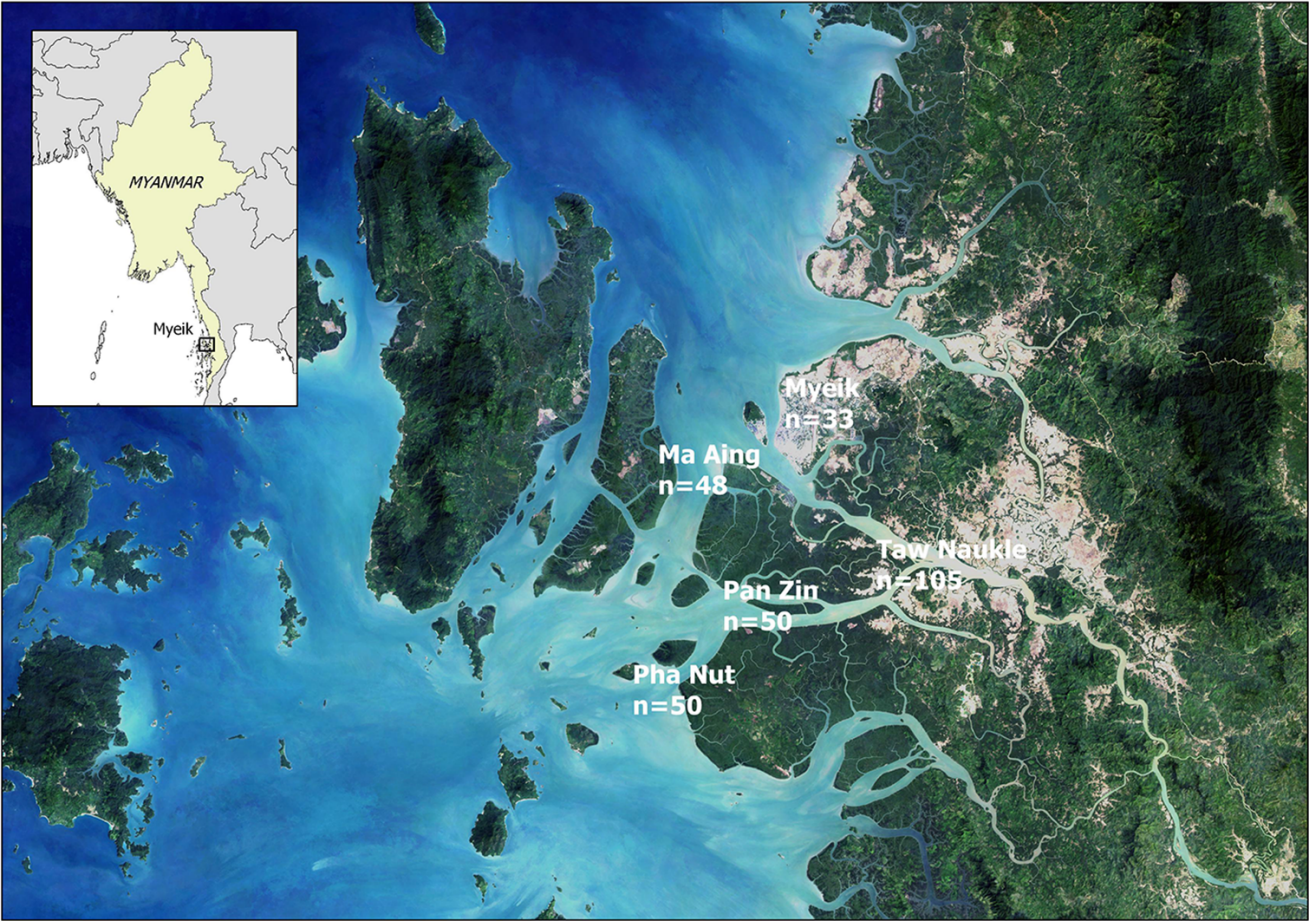
Studied area in the Mergui archipelago showing fishing communities included in this study and sample sizes.

In Myanmar, mud crabs are caught using baited traps, normally with sardine, which are set at the beginning of the high tide and retrieved at the end of the high tide (4–6 h later). The daily catch is normally stored in warehouses until export or transport to soft-shelled crab farms where the individuals are kept alive until they molt. While mud crabs were being prepared for storage, tissue samples, typically leg segments, were collected in four major crabbing communities of the Mergui archipelago in the Tanintharyi region and from a mud crab aggregating center in Myeik city during 2016–2017 (Fig 1). For each individual crab sampled, we recorded sex and size as internal carapace width (CW in mm). Tissue samples were dry-preserved in silica gel until processed for DNA barcoding. Genomic DNA was extracted using the DNeasy extraction kit (Qiagen, CA).

### DNA barcoding and PCR-based species identification

Fragments of 515 base pairs (bp) of the gene *COI* were amplified in 25 μL volume PCRs, consisting of 25 nM Tris-HCL, 25 nM NaCl, 2.5 mM MgCl2, 0.4 mM each dNTP and 0.08 mM of each primer: C/N 5' TT AAG TCC TAG AAA ATG TTG RGG GA 3' and mtd10 5' T TGA TTT TTT GGT CAT CCA GAA GT 3' [19], and 0.5 units of GoTaq Flexi (Promega). The cycling profile consisted of a denaturation step of 5 min at 94°C, followed by 35 amplification cycles of 30 sec at 52°C, 45 sec at 72°C and 30 sec at 94°C and a final elongation step of 5 min at 72°C. PCR products were purified with Exo-SAP-IT (Affymetrix, Inc., Santa Clara, CA, USA). PCR products were sequenced using BDX64 enhancing buffer (MCLAB, San Francisco, CA, USA) and the Big Dye Terminator v3.1 cycle sequencing (Applied Biosystems, Foster City, CA, USA) following the MCLAB BDX64 protocol and analyzed on an ABI 3730 DNA Analyzer (Applied Biosystems, Foster City, CA, USA) at the Smithsonian Laboratory for Applied Biology in Washington, DC. Sequences were checked for base call using the software Geneious 10.0.5 (http://www.geneious.com, [20]), and were aligned using CLUSTAL X [21]. All checked sequences were compared to homologous mt DNA *COI* sequences using two searchable databases: BOLD to search the Barcode of Life Data Systems and NCBI nucleotide repository with BLASTn on GenBank. We established confidence values for both BLASTn (identity > 98%) and BOLD (similarity > 98%) to ensure the reliable identification of species for each sample.

Samples resulting in an ambiguous BLASTn or BOLD score (>98%) for congeneric species, e.g. between *S*. *serrata* and *S*. *olivacea*, were amplified for species-specific *COI* fragments using a set of primers previously developed for PCR-based species identification (see [22]).

### Genetic characterization of the mud crab population

Unique mtDNA *COI* haplotypes were identified using DNAsp version 6 [23]. Haplotype diversity (*h*), nucleotide diversity (*π*), Tajima’s D and Fu’s Fs test for selective neutrality, and inbreeding coefficients (*F*_*ST*_ and *Φ*_*ST*_),were estimated using ARLEQUIN 3.5.2.2 [24]. Comparisons against conspecifics from neighboring countries were for 460 bp of *COI* overlapping sequence for samples from the Bay of Bengal (GenBank accession numbers KT964568—KT964599), Malaysia [25], India [22], and Bangladesh (GenBank accession numbers KX959992-KX959996), for *Φ*_*ST*_ analysis conducted in ARLEQUIN 3.5.2.2 [24]. In addition, DNA divergence between *S*. *olivacea* and *S*. *serrata* [26] was estimated as implemented in DNAsp version 6 [23].

Phylogenetic relationships among mtDNA *COI* haplotypes (515 bp) of *S*. *olivacea* were examined with a median-joining network (MJN) rooted with homologous sequences from *S*. *serrata* as an outgroup. The network was generated with the program NETWORK 4.5.1.0 [27]. Historical demography was investigated using mismatch distributions, the frequency distributions of pairwise differences between sequences [28]. An index of time since expansion expressed in mutational time (*τ* = *2μt*, where *μ* is the mutation rate/generation and *t* is time in generations), was estimated by a generalized non-linear least square approach using ARLEQUIN 3.5.2.2 [24].

## Results

### Genetic species identification

A total of 284 mud crabs out of 286 analysed were identified as *Scylla olivacea*, two samples could not be identified due to low DNA sequence quality. Partial sequences of 515 bp of the mtDNA *COI* gene were successfully obtained for N = 282 mud crabs, of these 208 sequences returned an unambiguous hit (>98% identity) to *S*. *olivacea* with BLASTn, only 74 sequences returned an ambiguous match between *S*. *olivacea* and *S*. *serrata* showing high percentage of identity ranging from 97.9–100% for both species when compared to existing barcodes in BLASTn. The BOLD search resulted in all sequences with an ambiguous match between these two species, also with a high percentage of similarity scores (S1 Table). The amplification of an additional fragment of 212 bp of the mtDNA *COI* gene by species-specific PCR resolved the species ambiguity and confirmed identification of 284 crabs as *S*. *olivacea*. Likewise, DNA divergence between *COI* sequences of *S*. *olivacea*, this study, and published data from *S*. *serrata* was higher than divergence between *S*. *olivacea* from two different localities (Table 1).

**Table 1:** DNA divergence of *COI* haplotypes between *Scylla olivacea* and *S*. *serrata*.

	*S*. *olivacea* Myanmar	*S*. *olivacea* Bay of Bengal	*S*. *olivacea* Malaysia	*S*. *serrata* *Indo-*West Pacific
*Scylla olivacea* **Myanmar**		3.752	3.997	**48.822**
*Scylla olivacea* **Bay of Bengal**	0.009		4.477	**69.736**
*Scylla olivacea* **Malaysia**	0.011	0.009		**69.448**
*Scylla serrata* **Indo-West Pacific**	**0.152**	**0.153**	**0.152**	

DNA Divergence of *COI* haplotypes (515 bp) between conspecific populations and between *Scylla olivacea* and *S*. *serrata*. Lower diagonal: Dxy = Average number of nucleotide substitutions per site between species, upper diagonal: average number of differences between groups. The large interspecific divergence between *S*. *olivacea* and *S*. *serrata* is shown in bold.

### Genetic characterization of the mud crab population

Among the 282 sequences analyzed of 515 bp, 40 *COI* haplotypes showing 39 variable sites were identified (Table 2). Overall, estimates of haplotype diversity were high, ranging from 0.719 to 0.791, and nucleotide diversities were low (Table 3).

**Table 2:** *Scylla olivacea COI* haplotype alignment and frequency by sampling location.

	Nucleotide position	Locations				
Haplotype	1111111112222222223333333333333333445 592225789990123455790000133445556789450 430363162584387035040369836891476246197	MK	TN	PZ	PN	MA
Hap1	CTTTAGATCCGCTCATAGACATTTCACCCATCTACTTGA	14	35	16	22	13
Hap2	..........A.............T..............	1	3		1	
Hap3	.......C...........................C...	1				
Hap4	...........................T.......C...		1			
Hap5	....GA...............C.............C.A.	10	38	16	13	17
Hap6	...............C........T..............	1				
Hap7	T..................................C...				2	
Hap8	....GA......C........C.............C.AG			1		
Hap9	....GA...........A.................C...			1		
Hap10	....GA....A..........C.............C.A.		3			
Hap11	....GA.......T.......C.............C.A.			1		
Hap12	..C.GA...............C.........T...C.A.					2
Hap13	..C.GA.............T.C.............C.A.	1	2	2		2
Hap14	....GA...............C.......T.....C.A.		1		1	
Hap15	....GA...............C.......T...G.C.A.				1	
Hap16	....GA...............C..........C..C.A.		1			
Hap17	....GA...............C.......G.....C.A.			1		1
Hap18	....GAG..............C.............C.A.		1			
Hap19	.C..GA............G..C.............C.A.	3	5	6	2	7
Hap20	.C.CGA.....T......G..C.............C.A.		1			
Hap21	.C..GA............G..C..T..........C.A.	1	1			
Hap22	**CC.CGA............G............C....TA.**					1
Hap23	.............................T.....C...		1	1		
Hap24	.....C...T...................T....TC...		1			
Hap25	..C....................C...........C...		2	1	1	
Hap26	........A..............C...........C...		2	1	2	1
Hap27	.........................G.........C...			1		
Hap28	.........TA........................C...		1			
Hap29	T..............................T...CC..				1	
Hap30	.........T.........................C...			1	1	
Hap31	............................T......C...		1			
Hap32	.................C.................C...			1		
Hap33	.............................G.....C...		1			
Hap34	..............G........C...........C...					1
Hap35	..........A...............T............				1	
Hap36	..............................C....C...				1	
Hap37	......G............................C...					1
Hap38	....GA..............GC.............C.A.					1
Hap39	.C..GA............G..CC............C.A.					1
Hap40	................G.................——					1

List of *COI* haplotypes (515 bp) derived for *Scylla olivacea* showing alignment, variable sites and frequency by locations. Location abbreviations: MK = Myeik, TN = Taw Naukle, PZ = Pan Zin, PN = Pha Nut, MA = Ma Aing.

**Table 3:** Genetic diversity, tests for neutrality and population expansion indexes.

	Myeik	Taw Naukle	Pan Zin	Pha Nut	Ma Aing	Overall
**π**	0.092	0.085	0.104	0.081	0.115	0.129
***h***	0.719	0.739	0.791	0.745	0.789	1
***tau***	4.852	4.469	5.197	4.121	6.188	4.5
**Expansion time (years)**	242 600	223 450	259 850	206 050	309 400	225 000
**D**	-0.712	-0.941	-0.206	-1.001	-0.119	-1.563
**D (*p*)**	0.252	0.19	0.48	0.169	0.54	0.032
**F_s_**	0.353	-6.315	-2.299	-3.75	-0.627	-25.722
**F_s_ (*p*)**	0.613	0.029	0.183	0.057	0.425	<0.001
**Mismatch *p***	0.04	<0.001	<0.001	0.02	<0.001	<0.001
**Raggedness index**	0.319	0.236	0.209	0.232	0.187	0.373

Genetic diversity indexes and tests for neutrality and population expansion based on mtDNA *COI* haplotypes (515 bp). Parameter symbols: π = nucleotide diversity, *h* = haplotype diversity, *tau* = divergence time, D = Tajima’s D, Fs = Fu’s Fs, p = p-value.

**Fig 2:**
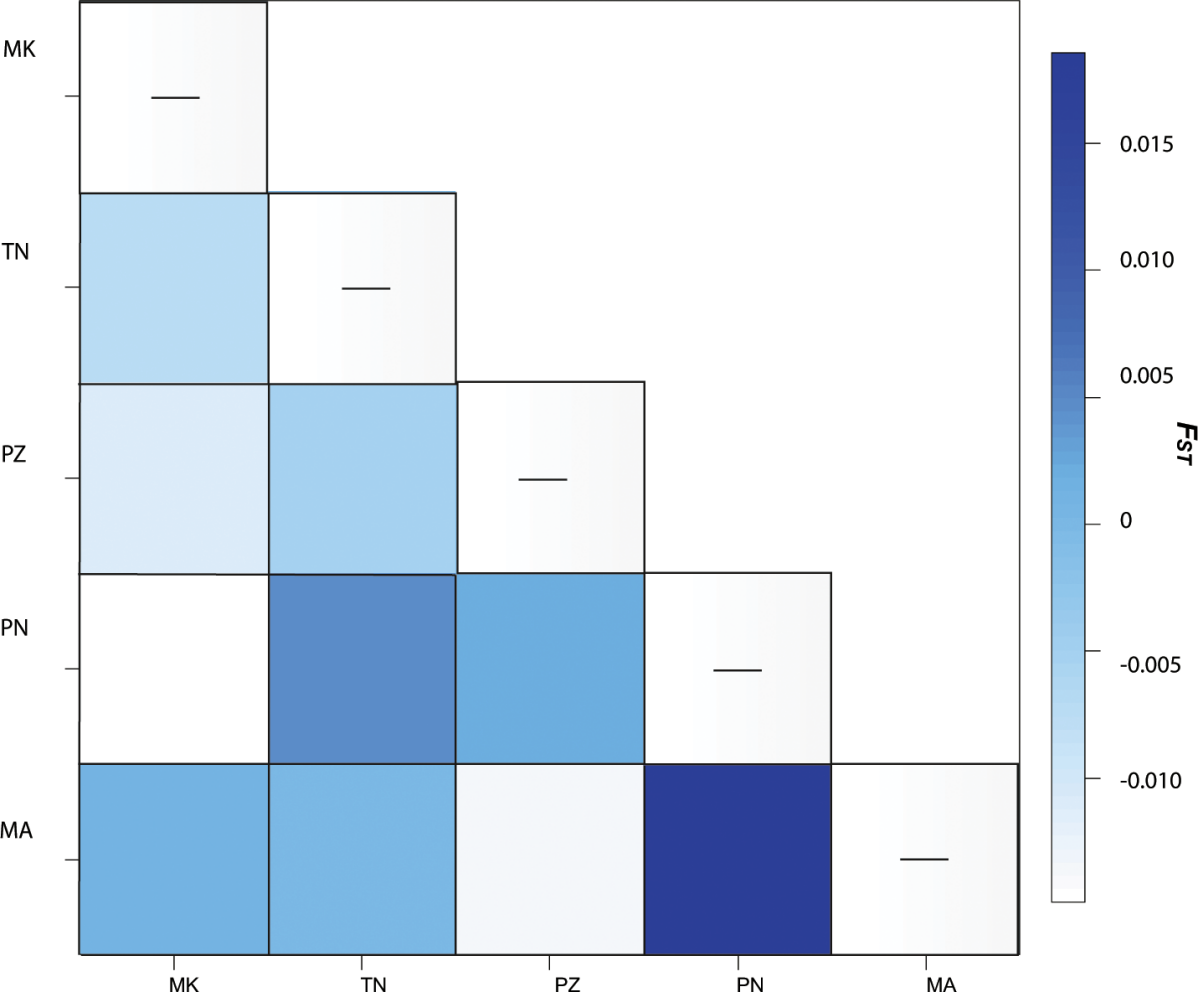
Fine-scale genetic differentiation estimated as *F*_*st*_ based on 515 bp of mtDNA *COI*. Heat-map of *F*_*ST*_ pairwise comparisons between the five mud crab sampling locations, MK = Myeik, TN = Taw Naukle, PZ = Pan Zin, PN = Pha Nut, MA = Ma Aing estimated as *F*_*st*_ values indicating broad gene flow between fishing communities within the Mergui archipelago.

**Fig 3:**
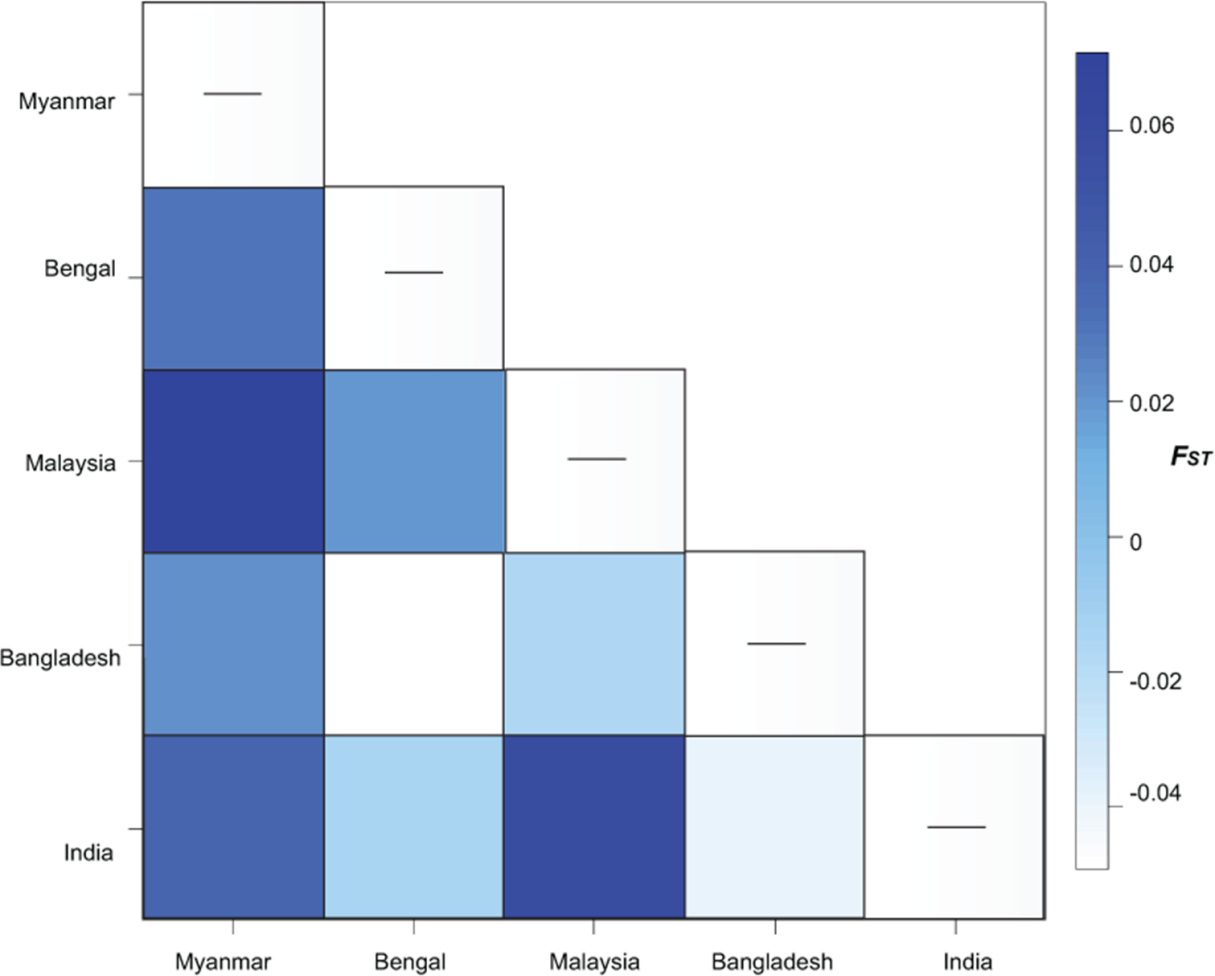
Regional genetic differentation estimated as *F*_*st*_ based on 460 bp of mtDNA *COI*. Heat-map of *F*_*ST*_ pairwise comparisons between populations of *Scylla olivacea* from Myanmar, Bay of Bengal, Malaysia, Bangladesh, and India indicating the restricted gene flow between neighbouring countries.

**Fig 4:**
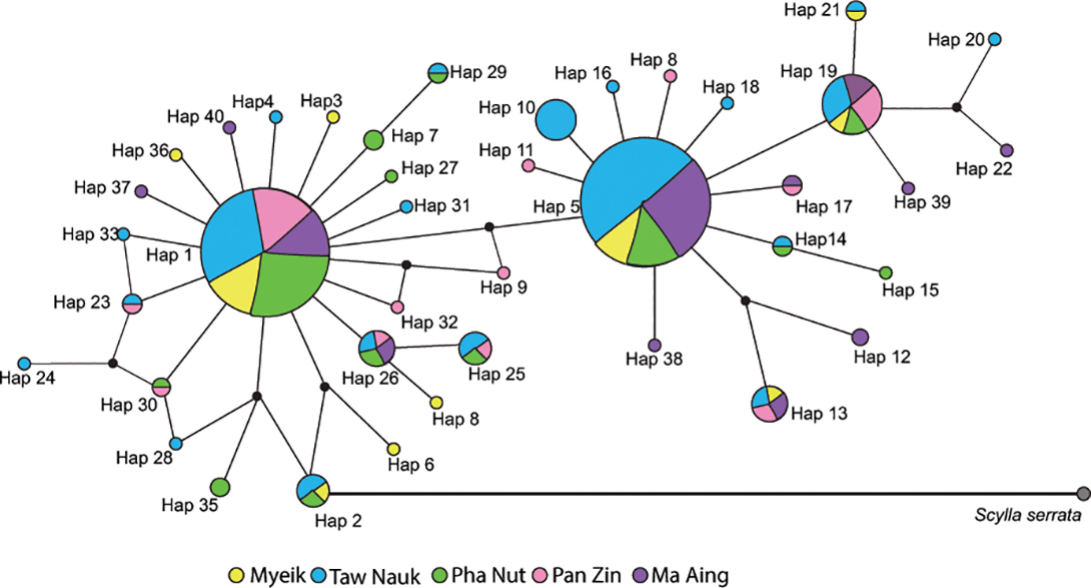
Median-joining network of the 40 COI haplotypes (515bp) of *Scylla olivacea*. Phylogenetic reconstruction of *Scylla olivacea COI* haplotypes. Circle diameter is proportional to haplotype frequency, and color are coded by location. *Scylla serrata*, gray circle, was used as the outgroup.

Fine scale haplotype geographic distribution and the number of shared haplotypes (Table 2) resulted in non-significant genetic differentiation (*F*_*ST*_ = -0.001, p = 0.531; *Φ*_*ST*_ = 0.526, p<0.00001) (Fig 2). Genetic structure analyses revealed significant differences in haplotype frequencies among haplotypes resulting in a highly significant fixation index (*F*_*ST*_ = 0.0296, p = 0.002). All *F*_*ST*_ pairwise comparisons between Myanmar and neighbor populations were significant, the strongest differentiation was against Malaysia (*F*_*ST*_ = 0.071, p<0.001) (Fig 3). All *Φ*_*ST*_ comparisons were low and non-significant. Both tests for neutrality, Tajima’s D and Fu’s Fs values were significant for pooled data suggesting evidence for an expansion signal, but were non-significant in all individual populations estimates (Table 3). The mismatch distribution showed a multimodal pattern and significant deviation from the model for expansion (Table 3, S1 Fig), suggesting population stability consistent with the haplotype phylogenetic reconstruction (Fig 4).

## Discussion

### Species identification

Our results found that *Scylla olivacea* was the only species harvested from the mangrove creeks across the Mergui archipelago, in southern Myanmar, as identified using DNA barcoding the first study of its kind in Myanmar’s marine fisheries. This is in accordance with the catch composition of the mud crab fisheries of Malaysia [25], Bangladesh [12], and Philippines [29], where *S*. *olivacea* is the only or the dominant species harvests from mangroves fishing grounds. The giant mud crab *S*. *serrata* was previously thought to be the most common species harvested across Southeast Asia, but more studies have revealed that *S*. *olivacea* is indeed the most common species [12,15,29]. Likewise, S. *olivacea* is the most common species found in the markets in Southeast Asia [2] and the most important crab for commercial culture in the Indo-West Pacific region leading to high prices in both local and international markets [30].

The absence of other *Scylla* congeneric species in this study does not mean that other species do not occur in the country. In close geographical proximity to Myanmar *S*. *olivacea* and *S*. *serrata* have been found in sympatry, e.g. in India [15,22,31], Thailand [13], and Bangladesh [12]. Our results indicate that *S*. *olivacea* is the main species harvested across the Mergui archipelago during the time of this study, but other species may occur or may be susceptible to other fishing gear. Fishers in the Tanintharyi region use only baited traps deployed in the mangrove intertidal zone, while in other countries fishers use different gears such as traps, lift and skate nets, fish in different habitats so may catch other *Scylla* species (see [29]). Habitat separation among congeneric species has been noticed, although not well explored, but suggests a strong preference of *S*. *olivacea* for the intertidal zone in mangrove habitats [3,29,32]. While *S*. *tranquebarica* was found commonly sub-tidally, and *S*. *serrata* occurs in lower temperatures [33].

*COI* species-specific fragments, (212 bp and 138 bp, for *S*. *olivacea* and *S*. *serrata*, respectively, [22]) confirmed the species identification using DNA barcode sequences generated in this study. Moreover, the estimates of high genetic divergence for *S*. *serrata* comparisons also support the species distinction (Table 3). Ambiguous results were returned when comparing our sequences against different public databases (GenBank and BOLD) suggesting that some *COI* sequences had been generated from species misidentified in previous studies when *S*. *serrata* was thought to be the dominant species. Therefore, caution must be taken when using public databases for species with controversial or recently reviewed taxonomy.

The correct identification of species being harvested in mud crab fisheries is the first step in developing effective and sustainable management practices. The biological information necessary to implement fishery regulations differs among species. For example setting a minimum size based on size of maturity needs to be linked to species specific reproductive biology. The current results can now help Myanmar develop fishery management and regulations in the Tanintharyi region focused on *Scylla olivacea*.

### Genetic characterization of Scylla olivacea

Mud crabs, *S*. *olivacea*, across the Tanintharyi region showed a high *COI* haplotype diversity and low nucleotide diversity (Table 2), which is explained by the numerous haplotypes, which differed from each other by only few mutations (see Table 1). This pattern of genetic diversity appears to be characteristic among crustacean species [34–36] including *S*. *olivacea* in Malaysia [25] and *S*. *serrrata* in East Africa [37]. This pattern suggests that *S*. *olivacea* has experienced genetic bottleneck events that caused the extinction of some haplotypes [38] and subsequent population expansion, thus enabling the retention of new mutations without sufficient time for accumulation of large differentiation among haplotypes [39]. Tajimas’s D and Fu’s *Fs* indexes were negative but non-significant for all populations, consistent with the excess of rare mutations observed, and consistent with a population at drift-mutation equilibrium. However, the negative and significant Fu’s *Fs* statistical value estimated for the pooled data provides strong evidence for past population expansion [40]. This is in agreement with mismatch distribution, the multimodal (including bimodal observed in the pooled data) distributions and the high raggedness indices estimated (Table 3) are characteristic of admixture of historically isolated populations, and a ragged distribution suggests that the lineage was widespread [28,41].

Moreover, the MJN star-like shaped phylogeny reconstruction (Fig 4), shows two well-defined lineages with a central haplotype, where Hap 1 and Hap 5 are the most probable ancestral haplotypes, surrounded by several haplotypes that show little base pair differences. This topology is typical for a population that has recently expanded in size from a small number of founders following a population bottleneck [42].

Overall, the distribution and frequency of the 40 *COI* haplotypes (Table 1), resulted in both negative and non-significant *F*_*ST*_ and *Φ*_*ST*_ values, indicative of no genetic structure among the five localities compared. These results suggests extensive gene flow among the sampled localities, likely driven by female reproductive behaviour [13,43], with migration up to 50 km from the mangroves out to sea to release larvae [44]. In addition, the dispersal capabilities could be extensive with the planktonic larva stage lasting up to four weeks [26,45], and strongly influenced by tide conditions and mixing floods during Monsoon season [46].

Despite the potential dispersal capabilities of early life stages of the species, the hypothesis of unrestricted gene flow among conspecifics from neighbouring countries was rejected, as estimated by the fixation index *F*_*ST*_ based on differences in haplotype frequencies. Pairwise comparisons showed a complex pattern of genetic differentiation, as expected given the fairly complex circulation of the Andaman Sea, where surface currents change seasonally depending on the northeast monsoon (December–February) and the southwest monsoon (June–September) [46,47]. Comparisons between Myanmar and Malaysia were the most differentiated (Fig 3), indicating a restricted gene flow regardless of their close geographic location, which is likely caused by the prevailing anti-cyclonic circulation during northeast monsoon and the weak transport through the Malacca Strait during southwest monsoon resulting in a limited mixing between water flowing through the straight and from the Myanmar coast [48]. A similar pattern of population structure among conspecifics was documented in *S*. *serrata* from East Africa, where ocean dynamics impose barriers to gene flow between geographically close populations, while promoting gene flow to further locations [37]. Likewise, the Asiatic mangrove, *Rhizophora mucroanta*, showed the most distinct genetic differentiation between the Andaman Sea and the Malacca Strait, which can be explained by the prevailing ocean currents in this region dictating the dispersal of propagules [48]. The less genetic differentiation among Myanmar, India, Bay of Bengal and Bangladesh might be maintained by the equatorial forcing and the local winds forcing the coastal circulations entering the Andaman Sea and around islands transporting waters from India into the north of the Andaman Sea [47].Ocean circulation and dynamics must be explored locally in greater detail to explain this complex pattern of genetic structure and to further investigate the geographical scale and dynamics of mud crab dispersion in this region. The fixation index *Φ*_*ST*_ was non-significant for all population pairwise comparisons, as expected given the low nucleotide diversity found in the species in this study and in Malaysia [25].

## Conclusions

These results suggest *Scylla olivacea* is the main or only species harvested in the mud crab fishery in the Mergui archipelago. Moreover, *S*. *olivacea* exhibits an extensive gene flow among the populations, but restricted gene flow among neighbouring countries Therefore, the population of *S*. *olivacea* in the Mergui archipelago may be susceptible to local depletion from overfishing as neighbouring countries may not contribute to replenish its fishing stocks, and so local management of the Myanmar mud crabbing is fundamental. These findings represent a critical baseline for further studies to assess the effect of fishing, overfishing, pollution, and habitat loss on the mud crab population and on the levels of genetic diversity, which is directly affected by the ability of the species to successfully adapt to natural and anthropogenic changes in the environment.

## Supporting information

S1 Table Results for *Scylla* species identification from comparisons to databases in BLASTn and BOLD. Showing only results for unique haplotypes (n = 40).
(PDF)

S1 Fig Mismatch distribution for each five localities in this study, and overall five populations. MK = Myeik, TN = Taw Naukle, PZ = Pan Zin, PN = Pha Nut, MA = Ma Aing.
(PDF)
