# Supplementary material for: Genetic characterization of the artisanal mud crab fishery in Myanmar
Source: PLoS One. 2018 Sep 28;13(9):e0204905. doi: 10.1371/journal.pone.0204905 (PMC6161904; doi:10.1371/journal.pone.0204905)
Supplement: S1 Table — Showing only results for unique haplotypes (n = 40). (PDF) [file pone.0204905.s001.pdf]

S1 Table. Results for *Scylla* species identification from comparisons to databases in BLASTn and BOLD. Showing only results for unique haplotypes (n=40).

Field-ID	Haplotype	**BLASTn** best ID	ID %	**BOLD** best ID	SIM %
Ss51	H1	*Scylla serrata/olivacea*	99.8	*Scylla serrata/olivacea*	100
Ss284	H2	*Scylla olivacea*	100	*Scylla serrata/olivacea*	100
Ss135	H3	*Scylla olivacea*	99.6	*Scylla serrata/olivacea*	100
Ss164	H4	*Scylla serrata/olivacea*	99	*Scylla serrata/olivacea*	100
Ss168	H5	*Scylla serrata/olivacea*	100	*Scylla serrata/olivacea*	99.29
Ss108	H6	*Scylla olivacea*	99.8	*Scylla serrata/olivacea*	100
Ss247	H7	*Scylla olivacea*	97.9	*Scylla serrata/olivacea*	99.29
Ss200	H8	*Scylla olivacea*	99.8	*Scylla serrata/olivacea*	99.29
Ss206	H9	*Scylla olivacea*	98.9	*Scylla serrata/olivacea*	99.29
Ss152	H10	*Scylla olivacea*	99.8	*Scylla serrata/olivacea*	99.29
Ss223	H11	*Scylla olivacea*	99.6	*Scylla serrata/olivacea*	99.29
Ss314	H12	*Scylla olivacea*	99.6	*Scylla serrata/olivacea*	98.58
Ss52	H13	*Scylla olivacea*	99.8	*Scylla serrata/olivacea*	98.58
Ss145	H14	*Scylla olivacea*	100	*Scylla serrata/olivacea*	99.29
Ss265	H15	*Scylla olivacea*	99.3	*Scylla serrata/olivacea*	99.29
Ss144	H16	*Scylla olivacea*	99.8	*Scylla serrata/olivacea*	99.29
Ss324	H17	*Scylla olivacea*	99.8	*Scylla serrata/olivacea*	99.29
Ss68	H18	*Scylla olivacea*	99.8	*Scylla serrata/olivacea*	99.29
Ss178	H19	*Scylla olivacea*	98.3	*Scylla serrata/olivacea*	98.58
Ss80	H20	*Scylla serrata/olivacea*	99.8	*Scylla serrata/olivacea*	97.87
Ss157	H21	*Scylla olivacea*	99.3	*Scylla serrata/olivacea*	98.58
Ss319	H22	*Scylla olivacea*	99.8	*Scylla serrata/olivacea*	97.87
Ss186	H23	*Scylla serrata/olivacea*	99	*Scylla serrata/olivacea*	100
Ss57	H24	*Scylla olivacea*	99.4	*Scylla serrata/olivacea*	100
Ss190	H25	*Scylla olivacea*	99.8	*Scylla serrata/olivacea*	99.29
Ss268	H26	*Scylla olivacea*	99.6	*Scylla serrata/olivacea*	100
Ss220	H27	*Scylla olivacea*	99.8	*Scylla serrata/olivacea*	100
Ss87	H28	*Scylla olivacea*	100	*Scylla serrata/olivacea*	100
Ss153	H29	*Scylla olivacea*	99.6	*Scylla serrata/olivacea*	99.29
Ss230	H30	*Scylla olivacea*	99.8	*Scylla serrata/olivacea*	100
Ss156	H31	*Scylla olivacea*	99.6	*Scylla serrata/olivacea*	100
Ss203	H32	*Scylla olivacea*	99.8	*Scylla serrata/olivacea*	100
Ss146	H33	*Scylla olivacea*	99.8	*Scylla serrata/olivacea*	100
Ss320	H34	*Scylla olivacea*	99.1	*Scylla serrata/olivacea*	100
Ss274	H35	*Scylla olivacea*	99.1	*Scylla serrata/olivacea*	100
Ss278	H36	*Scylla olivacea*	99.6	*Scylla serrata/olivacea*	100
Ss297	H37	*Scylla olivacea*	99.4	*Scylla serrata/olivacea*	100
Ss335	H38	*Scylla serrata/olivacea*	99.4	*Scylla serrata/olivacea*	99.29
Ss336	H39	*Scylla olivacea*	99.1	*Scylla serrata/olivacea*	98.58
Ss250	H40	*Scylla serrata/olivacea*	99.7	*Scylla serrata/olivacea*	100
