# Supplementary material for: Genetic characterization of the artisanal mud crab fishery in Myanmar
Source: PLoS One. 2018 Sep 28;13(9):e0204905. doi: 10.1371/journal.pone.0204905 (PMC6161904; doi:10.1371/journal.pone.0204905)

S1 Fig. Mismatch distribution for each five localities in this study, and overall five populations. MK=Myeik, TN= Taw Naukle, PZ= Pan Zin, PN= Pha Nut, MA= Ma Aing.

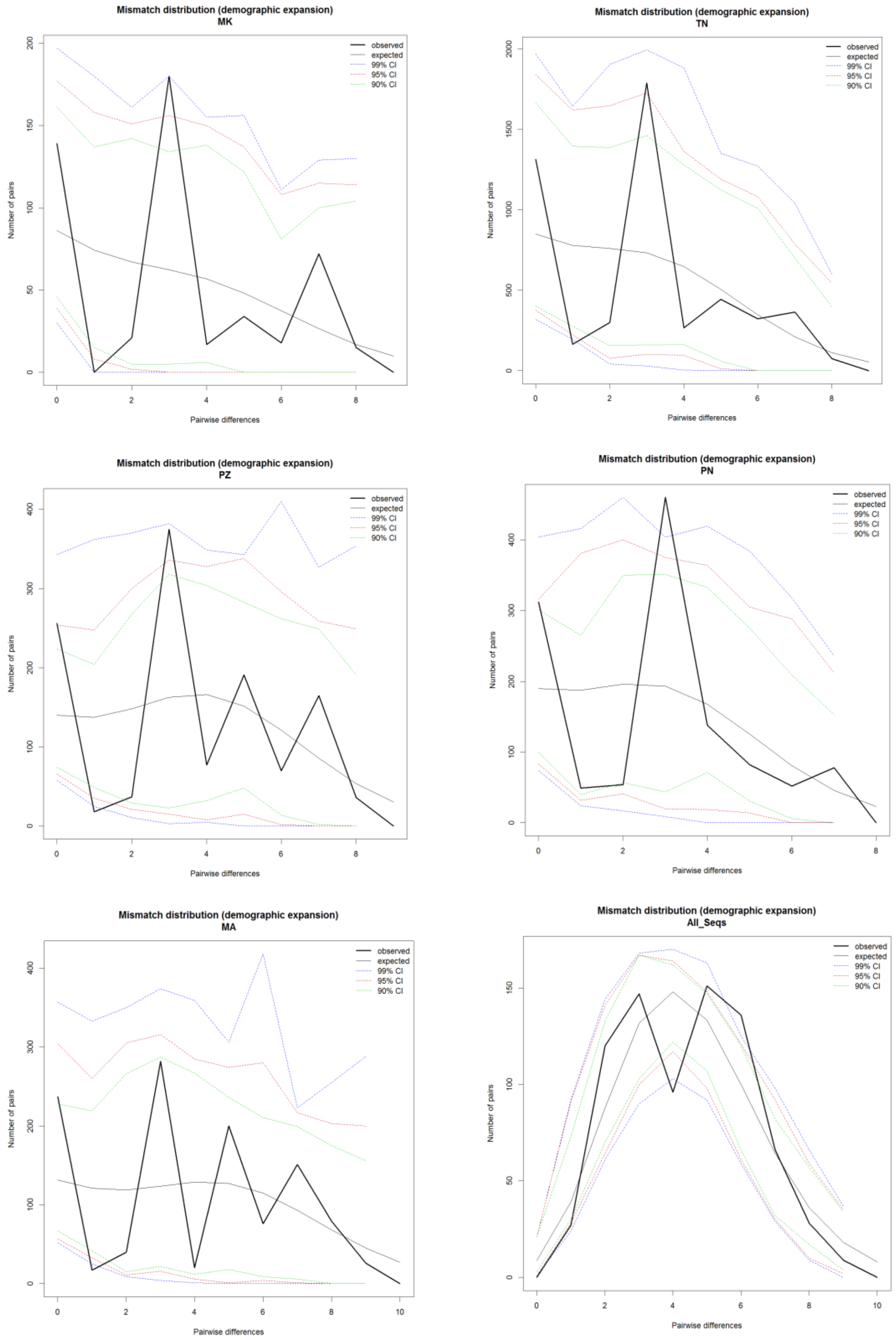

Supplement: S1 Fig — MK = Myeik, TN = Taw Naukle, PZ = Pan Zin, PN = Pha Nut, MA = Ma Aing. (PDF) [file pone.0204905.s002.pdf]
